# Gene Gangs of the Chloroviruses: Conserved Clusters of Collinear Monocistronic Genes

**DOI:** 10.3390/v10100576

**Published:** 2018-10-20

**Authors:** Phillip Seitzer, Adrien Jeanniard, Fangrui Ma, James L. Van Etten, Marc T. Facciotti, David D. Dunigan

**Affiliations:** 1 Department of Biomedical Engineering, One Shields Ave, University of California, Davis, CA 95616, USA; phillipseitzer@gmail.com; 2 Genome Center, One Shields Ave, University of California, Davis, CA 95616, USA; 3 Proteome Software, Portland, OR 97219, USA; 4 Nebraska Center for Virology, Morrison Research Center, University of Nebraska, Lincoln, NE 68583-0900, USA; fangrui.ma@gmail.com (F.M.); jvanetten1@unl.edu (J.L.V.E.); 5 Department of Plant Pathology, Plant Science Hall, University of Nebraska, Lincoln, NE 68583-0722, USA

**Keywords:** gene gang, *Chlorovirus*, synteny, collinearity, monocistronic, gene neighborhood, functional annotation, operon, mosaicism, genomic island, genomic metabolon

## Abstract

Chloroviruses (family *Phycodnaviridae*) are dsDNA viruses found throughout the world’s inland waters. The open reading frames in the genomes of 41 sequenced chloroviruses (330 ± 40 kbp each) representing three virus types were analyzed for evidence of evolutionarily conserved local genomic “contexts”, the organization of biological information into units of a scale larger than a gene. Despite a general loss of synteny between virus types, we informatically detected a highly conserved genomic context defined by groups of three or more genes that we have termed “gene gangs”. Unlike previously described local genomic contexts, the definition of gene gangs requires only that member genes be consistently co-localized and are not constrained by strand, regulatory sites, or intervening sequences (and therefore represent a new type of conserved structural genomic element). An analysis of functional annotations and transcriptomic data suggests that some of the gene gangs may organize genes involved in specific biochemical processes, but that this organization does not involve their coordinated expression.

## 1. Introduction

Viruses included in the genus *Chlorovirus*, family *Phycodnaviridae*, encode up to 416 computationally predicted protein sequences (CDSs) and contain many more open reading frames (ORFs). Chloroviruses infect certain unicellular eukaryotic, chlorella-like green algae [1,2,3]. Known chlorovirus hosts, which are normally endosymbionts and often referred to as zoochlorellae, are associated with either the protozoan *Paramecium bursaria*, the coelenterate *Hydra viridis*, or the heliozoon *Acanthocystis turfacea.* Three such zoochlorellae are *Chlorella variabilis* NC64A (formerly named *Chlorella* NC64A—its viruses are called NC64A viruses), *Chlorella heliozoae* (formerly named *Chlorella* SAG 3.83—its viruses are called SAG viruses), and *Micractinium conductrix* (formerly named *Chlorella* Pbi—its viruses are called Pbi viruses).

Genomes of 41 viruses collected from five continents and infecting the three different hosts have been sequenced, assembled, and annotated [4]. Collectively, the 41 *Chlorovirus* genomes encode 632 protein families, whereas any given virus has 319 to 416 predicted CDSs. Of these 632, 155 protein families (with 188 members) are found in all chloroviruses, referred to as “core proteins”, and many of these core proteins contribute central functions for virus replication, including: (1) virus entry; (2) DNA replication; and (3) regulation of gene expression. However, many of the core proteins have unknown functions and have little or no significant resemblance to proteins in existing databases (Table S1: Comprehensive table of gene annotations—Table_S1.xlsx). 

Though gene content tends to be conserved within the chloroviruses, overall gene order tends to be scrambled among viruses that infect the three hosts [4]. Nevertheless, we noticed that certain genes tended to be genomically localized next to one another in many of the viruses, independent of their host range. This observation led to a broader examination of the protein families using the full set of 41 sequenced and annotated chloroviruses where we evaluated whether certain genes tend to cluster together with a common synteny. When we use the term “synteny” or “syntenic,” we mean the physical distance of the genes of interest remain in close proximity, but are not constrained by strandedness or positional relationship among genes. Furthermore, co-segregating gene clusters were investigated for the potential to contribute to metabolic or physiological functions essential to virus success, including the functions (and putative functions) of multiple coordinated proteins. 

## 2. Materials and Methods

### 2.1. Assembly of the Chlorovirus Dataset

Annotated *Chlorovirus* genomes were retrieved from NCBI. Protein families and phylogenetic trees were retrieved from supplementary information in the manuscript by the authors of [4]. Phylogenetic trees and genomic segments were evaluated using the genomic context and visualization tool JContextExplorer [5].

### 2.2. Determination of Single-Copy Core Gene Pair Distances

We encourage the reader to consult a copy of Figure S4 while reading this section.

Single Copy Core Genes (SCCGs) are defined as a set of homologous genes that are each present only once in a set of viral genomes. Translated coding regions in all 41 viruses were extracted, and homologous proteins were identified and clustered into homology groups previously [4]. Homology clusters were analyzed using R [6] to determine clusters for which only a single cluster member could be assigned to each of the 41 genomes and formed the set of SCCGs.

#### 2.2.1. Strictly Constrained Bin Analysis

All SCCG pairs in a *Chlorovirus* genome were sorted into bins based on the distance separating gene centers. Binning began with a SCCG pair distance of 0 kbp, separated by 1 kbp, increasing every 1 kbp until 280 kbp (bin 1 contains all SCCG pairs with center-to-center distances of 0–1 kbp; bin 2 contains all SCCG pairs with center-to-center distances of 1–2 kbp, increasing every 1 kbp to 280 kbp). The analogous SCCG pair was recovered in all other genomes, and the number of times these pairs were located within 1 kbp ± 250 bp of the original distance was tabulated. The percent of analogous SCCG pairs for which distance was conserved in 100%, 90%, 80%, and 70% of other genomes was also evaluated.

#### 2.2.2. Growing Bin Analysis

The analysis of conservation of SCCG distances in the context of growing bins was conducted for all possible pairwise genomic comparisons. By contrast to the constrained bin analysis, distance bins used for comparison of analogous SCCG pairs in the growing bin analysis were allowed to incrementally grow by 1 kbp up to a maximum bin size of 280 kbp (bin 1 contains all SCCG pairs with center-to-center distances of 0–1 kbp; bin 2 contains all SCCG pairs with center-to-center distances of 0–2 kbp, increasing every 1 kbp to 280 kbp).

### 2.3. Determination of Phylogenetic Distances

Phylogenetic distances were computed from the phylogeny presented by Jeanniard et al. (2013) [4] (File S2: Newick formatted phylogeny of chloroviruses—File_S2.nwk). The Jeanniard phylogeny is a maximum likelihood tree of chloroviruses based on a concatenated alignment of 32 core protein families. The phylogenetic tree was computed using the WAG + G + I substitution model. The R package ape was used to read the Newick file [7], a phylobase was used to convert the tree file for further analysis [8], xlsx was used to read and write Microsoft Excel files [9], and adephylo was used to compute a given distance between a set of tips in the phylogeny [10].

### 2.4. Identification of Gene Gangs

Gene gangs were identified using a set of scripts (see File S3: scripts used to calculate gene gangs—File_S3.zip) written in Java. A graphical overview of the method is provided in Figure S4: An overview of the method used to determine gene gangs—Figure_S4.png.

Homology cluster IDs (clusters of homologous genes) were associated with genomic features in the 41 chloroviruses [4]. Examining these clusters, we identified cases where a single cluster ID was associated with multiple paralogs found frequently in multiple copies in the *Chlorovirus* genomes. To avoid any ambiguity or confusion associated with resolving genomic contexts of multiple paralogs, we classified any cluster that was identified more than 80 times in all 41 chloroviruses as a “large cluster.” We refer to all non-large clusters as “seed clusters”.

All seed clusters were used to form “cluster gangs”. This was done by first considering the localization of a given seed cluster in each individual *Chlorovirus* genome and the location of each homolog(s) member of a homology cluster in its respective genome. In the event that multiple homologs belonging to a single cluster were identified in a single *Chlorovirus* genome, all genomic features corresponding to a seed cluster were recovered from that genome. For each of these “seed features”, all other features in that organism’s genome with a center-to-center feature distance to the seed feature of 3500 base pairs or less were identified. These “collinear features” included genetic features that were both upstream and downstream of the seed feature, and could be on either strand. The seed feature and all identified collinear features were organized together and reported as a single “genomic grouping”. All of the genomic groupings identified from a seed cluster in each of the individual *Chlorovirus* genomes were collected and organized into a “cluster gang” (indicated as “Core genes within the collinear window” in Figure S4).

Typically, only one seed feature was identified in each individual *Chlorovirus* genome. However, there were a few cases in which a single gene was split into two proximal, collinear genes, perhaps because of errors in the structural gene annotation process. This resulted in what typically appeared as a single seed feature in most genomes being identified multiple times in another individual *Chlorovirus* genome. In these cases, genomic groupings were formed considering all genes found from the gene-to-gene center of each instances of the apparently redundant seed cluster—in effect, the process described in the previous paragraph was conducted for each apparently redundant individual seed feature, and the results were combined. Had multiple-genome paralogs not been excluded from the seed clusters as a first step (i.e., excluding the “large clusters”), genomic groupings for an organism might otherwise contain multiple non-collinear populations of collinear features (e.g., two paralogs localized on opposite ends of the genome pick up two distinct populations of collinear features surrounding them), complicating the interpretation of the results.

This initial set of cluster gangs were filtered to only contain those whose seed cluster was identified frequently in the chloroviruses. Specifically, only those cluster gangs featuring member seed clusters that occurred in at least 34 of the 41 (82.9% of the time) chloroviruses were retained (indicated as “Conserved genes in the collinear window” in Figure S4).

The set of filtered cluster gangs were next transformed into “gene gangs.” While cluster gangs are cluster number-centric, gene gangs are, by contrast, gene-centric. We required that a genomic feature not exist in more than one gene gang, which is not required of cluster gangs. If two cluster gangs were ever found to overlap in the cluster IDs featured among all genomic features in those cluster gangs, these cluster gangs were merged together into a single cluster gang. The new set of cluster gangs were then examined, and the process of examination and merging repeated itself until a set of gene gangs was produced where no genomic feature existed in more than one gene gang.

The process of forming gene gangs from cluster gangs is guaranteed to converge. However, it may converge to a single large gang if care is not taken to calibrate the set of parameters used in this process to the overall collinear similarity of the genomes examined. For these 41 chloroviruses, choosing the parameters max genomic distance, min organism prevalence (3500 bp and 34/41, respectively) is sufficient to avoid the case of converging to a single large gene gang containing all genomic features in all genomes.

The set of gene gangs is then adjusted to correct for any errors that might have been introduced by the exclusion of large clusters from the cluster gang formation step (described earlier). Specifically, there may be cases where multiple paralogs found in the same individual *Chlorovirus* genome should be included in a gene gang if the paralogs are sometimes located at the very edge of a genomic grouping, and not always incorporated into the genomic grouping by the cluster gangs associated with other genomic features. An example of this occurs in Figure 4, the “Gene Gang 7: Virus Entry” in PBCV-1, the green hypothetical protein.

For each gene gang, all clusters associated with all genomic features contained in a gene gang (in all genomes) were identified. We call this set the “gang cluster IDs.” For every genomic feature in every individual *Chlorovirus* genome, all genomic features were identified with a center-to-center distance of less than or equal to 3500 bp. These features may be either upstream or downstream of the feature of interest, and may exist on either strand. If any of these identified features had a cluster ID that was in the set of gang cluster IDs, and did not already exist in the gang, this feature was noted. After this process was carried out for all genomic groupings in all individual *Chlorovirus* genomes in the gene gang, if the genomic feature had been identified in at least 15 genomes (we may call this parameter the “paralog correction genome prevalence threshold”), the genomic feature was added to the gene gang, and to all genomic groupings where it was discovered.

After this adjustment step was performed, gene gangs were filtered once more, retained only if they contained three or more gang cluster IDs, and genomic features from all 41 chloroviruses. This completed the gene gang formation process, yielding a set of completed gene gangs for the chloroviruses.

Each gene gang contains a set of “gang cluster IDs,” which refers to the set of cluster IDs identified among all genomic features in the gene gang. The “ruliness” of a particular cluster ID is the proportion of genomes that contain at least one genomic feature with that cluster ID. We may filter gene gangs based on a provided “ruliness” threshold, which would remove all cluster IDs and corresponding genomic features from a gene gang with a ruliness value less than the submitted value. In our data, all cluster IDs have a ruliness value of at least 34/41 (0.829), because this was specified as a parameter in the gene gang formation process (“minimum genome prevalence”). The maximum ruliness value is 1.00, indicating that every organism in a gene gang contains at least one genomic feature with the cluster ID.

When speaking about the “ruliness” of a particular gene gang, this may take on two different meanings, depending on the context: (1) “gene gang X, with ruliness of Y” indicates that the minimum cluster ID ruliness of the gang cluster IDs of gene gang X is Y; and (2) “gene gang X, with ruliness of Y” indicates that gene gang X was subjected to a ruliness filter of Y, which removes any cluster IDs and corresponding genomic features of the gene gang less than Y. For example, suppose gene gang X contains three cluster IDs, <A, B, C>, with ruliness numbers <1.00, 1.00, 0.90>. We would say gene gang X has a ruliness of 0.90. Gene gang X at a ruliness of 1.00 would only consist of two cluster IDs, <A, B>, because cluster ID C was filtered out (its ruliness value of 0.90 did not pass the filter).

When we formed the gene gangs from our set of 41 chloroviruses, the following parameters were used:

max genomic distance: 3500 bp

min genomic prevalence: 34/41 genomes

paralog correction genome prevalence threshold: 14/41 genomes

The output of this process is available for view, in JContextExplorer by selecting “chloroviruses” under the “Popular Genome Set” menu item. The set of gene gangs are available in JContextExplorer as “GeneGangs_Strict,” which has a ruliness filter of 1.00 applied, and as “GeneGangs_Unruly,” which has no ruliness filter applied.

### 2.5. Functional Annotation Methods

The NC64A *Chlorovirus* PBCV-1 is the type member of the genus and has been evaluated most extensively, including: (1) genomic sequencing (and re-sequencing), assembly, and functional annotation of predicted proteins [4,11]; (2) transcriptional profiling [12,13]; (3) evaluation of specific gene features [14]; and (4) proteomic analyses of the intact mature virion [11]. To infer the potential for integrated gene gang functions, subsequent protein function annotations were made using tools from the NCBI, including RefSeq (“product,” “miscellaneous features,” and “note”). Additionally, conserved domains (http://www.ncbi.nlm.nih.gov/refseq/) were incorporated, as well as the primary and secondary matches of the hidden Markov model databases in SCOP [15,16]. These features were compiled with respect to each of the protein associations in a gene gang. Inferences of gang member functionality are listed as “inferred function.” In some cases, a reasonable integrated gang function can be predicted; for example, gene gang 12 appears to be operating in the realm of DNA replication, recombination and repair, with four of five members associated with DNA metabolism. In some cases, the inferred function remains a mystery.

## 3. Results

### 3.1. Chlorovirus Genomic Stability

**Figure 1:**
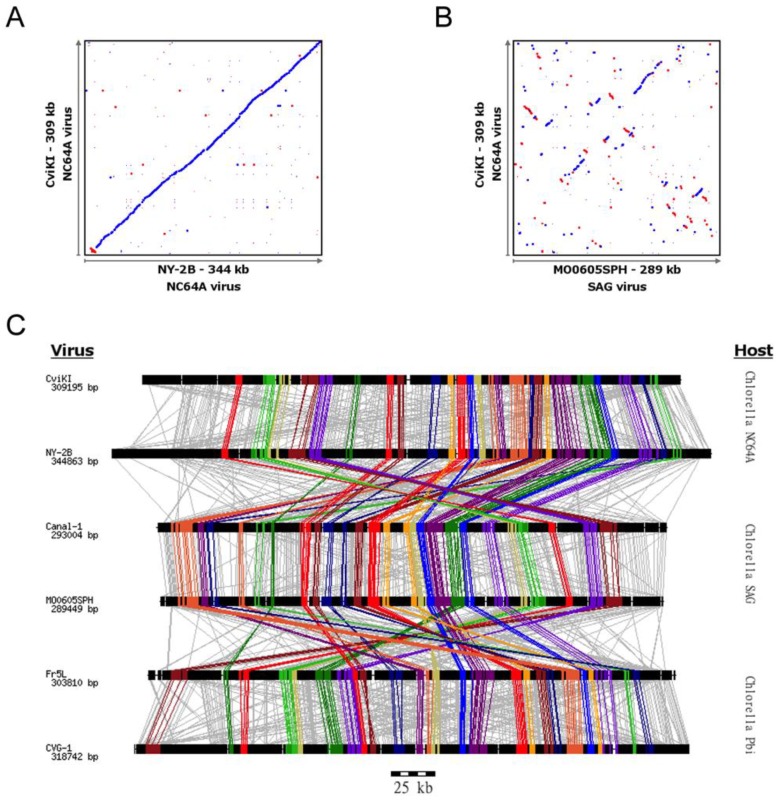
Gene dot plots and gene alignments across chlorovirus host types. Dot-plot alignments of two *Chlorovirus* genomes infecting the same (**A**) or different (**B**) host (blue indicates common genomic orientation, red indicates opposite genomic orientation). Each dot represents a protein match between the two viruses (BLASTp expect value < 1 × 10^−5^). (**C**) Schematic representation of six *Chlorovirus* genomes from three different hosts. Each genome is depicted as a black line with genic regions represented by black boxes. Lines are connecting genes from the same family of homologous proteins (for clarity purpose, the six bigger families were removed). The colored set of genes and lines represents the 25 gene gangs. While viruses that infect the same host often demonstrate good conservation of synteny (**A**), synteny is poorly conserved across virus types (**B**). Despite generally poor syntenic conservation, gene-centric alignments suggest the existence of some conserved collinear blocks (**C**).

The conservation of genomic structure in the chloroviruses varies depending on evolutionary distance. A high degree of collinearity of the genes throughout the genome is observed when comparing genomic synteny within a set of viruses that infect the same host. For example, this can be demonstrated with dot-plot analyses of full genomes comparing two NC64A viruses (Figure 1A). All of the analyzed NC64A and SAG viruses have a high level of synteny when compared within the type [4]. All of the analyzed Pbi viruses, with the exception of NE-JV-1, also have a high degree of synteny within the type. At this point, it may not be possible to disentangle genome structure as a proxy for evolutionary distance, from host range. However, very little large-scale synteny conservation is observed when comparing the genome architecture of viruses that infect different hosts (Figure 1B, comparing a NC64A virus to a SAG virus), despite relatively high conservation of gene content. These qualitative comparisons suggest that the evolution of these genomes involves a “shuffling” of the genetic material whose mechanism is yet to be completely understood.

Closer inspection of the dot-plots (e.g., Figure 1B,C), genome alignments (Figure 1) and manual browsing of the *Chlorovirus* genomes suggests there might be sets of the 155 core genes [4]. These core genes are conserved across all genomes, whose relative proximity to one another is conserved even if their order, strandedness or neighboring genes are not as tightly conserved. That is, certain genes appear to “hang out” together. To explore this apparent organizational pattern a systematic analysis of conservation of pairwise gene distances was performed.

### 3.2. Conservation of Distance between Single Copy Core Genes

We sought to determine the degree to which distances between pairs of genes were conserved in the genomes of the chloroviruses. We proposed that if an evolutionary pressure exists to constrain the distance between genes, a systematic examination of center-to-center distances between gene pairs could reveal the presence of conservation that is greater than would be expected by chance. To determine if such a distance (or distances) exists, we carried out two different analyses: the strictly constrained bin analysis and the growing bin analysis (see Methods).

The strictly constrained bin analysis determined how often there is strict conservation of distance between pairs of genes across the chloroviruses. For example, if genes A and B are 2.2 kbp apart in genome 1 (the seed genome), how often are the homologs of genes A and B also about 2.2 kbp apart in the other genomes? This question examines whether a specific distance between genes is conserved (e.g., the specific distance between genes is highly constrained). By contrast, the growing bin analysis determined how frequently an observed distance between specific pairs of genes exists in the same distance “size bin” among all the viral genomes. For example, if genes A and B are 2.2 kpb apart in genome 1, and therefore fit into a “distance size bin” of 0–5 kbp, how frequently are homologs for genes A and B located in the same “distance size bin” of 0–5 kpb in the other genomes?

In both analyses, we make the first-order assumption that each gene represents an independent shuffling unit and make pairwise distance comparisons between the set of orthologous genes that are present in all genomes in single copy. We termed this gene set the “Single Copy Core Genes” (SCCGs) set. The 41 *Chlorovirus* genomes contain 125 SCCGs.

#### 3.2.1. Results of Strictly Constrained Bin Analysis

**Figure 2:**
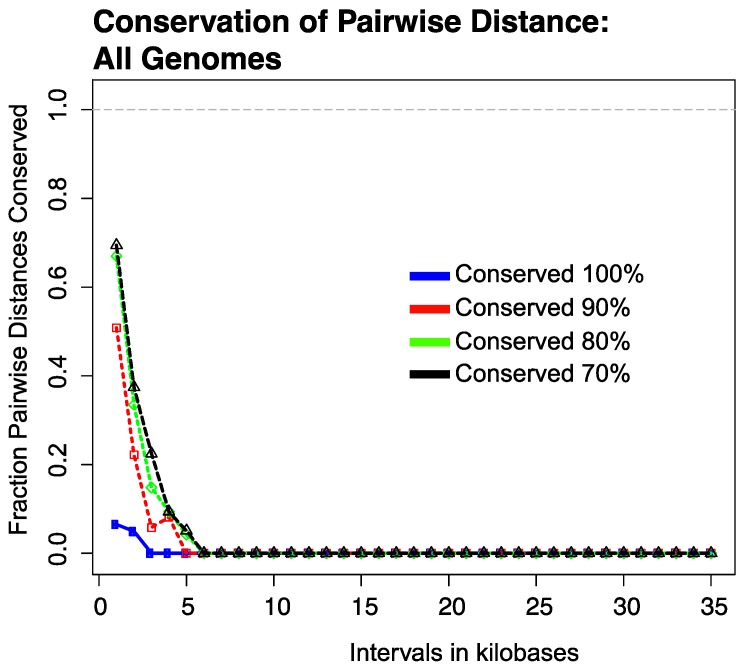
Analysis of pairwise conservation of Single Copy Core Gene (SCCG) distances in 1000-bp bins analyzed for all *Chlorovirus* genomes. The SCCG set determined for all genomes is used to determine the degree to which distances between specific pairs of SCCG are conserved across all genomes. Distances are considered to be conserved if they fall within the same 1000-bp size bin (e.g., 2000–3000 bp, 5000–6000 bp, etc). Each data point on the *x*-axis represents the upper bound of the bin size. The first data point (*x* = 1) represents an analysis of pairwise distances in a search genome between 0 and 1000 bp while the tenth data point (*x* = 10) represents an analysis of pairwise distance in a search genome between 9000 and 10,000 bp. Specific pairwise distances (assigned to a unique pair of SCCGs) in a search genome are searched against all other genomes. The y-axis reports the fraction of the pairwise distances considered in the size bin in question that were conserved in 100% (blue line), 90% (red line), 80% (green line), and 70% (black line) of all other genomes, respectively.

In this analysis, all possible pairwise center-to-center distances between SCCGs were systematically queried against distance intervals, each “bin” increasing by 1 kbp (e.g., probed intervals equaled 0–1 kbp, 1–2 kbp, 2–3 kbp, etc.). For example, in this analysis if the center-to-center distance between hypothetical SCCGs A and B is found to be 1243 bp, we ask: in what fraction of the remaining genomes is the distance between ortholog SCCGs A and B also between 1000 and 2000 bp ± 250 bp? The 250 bp term provides a small buffer against overly strict distance conservation. The results of this analysis are presented in Figure 2. This plot presents the percent of gene pairs whose distance is conserved within the 1 kbp ± 250 bp buffer described above. Four curves are shown, each representing the percent (100%, 90%, 80%, 70%) of viral genomes for which the pairwise distance between two specific genes was found to be conserved. This analysis, therefore, demonstrates that across all genomes, conservation of strict pairwise distance falls to nearly zero percent when SCCGs are greater than 5 kbp apart, even when this distance constraint is not required to be met in 30% of the genomes. This suggests that the distance between any pair of genes may only be constrained to stay within a strictly constrained bin size at short distances.

We hypothesized that the conservation of strict distances between gene pairs was likely due to the conservation of collinear blocks of genes in closely related genomes. To examine the influence of evolutionary distance on the conservation of strict pairwise distances, the analysis described in the previous paragraph was repeated using genomes both within and across the viral taxonomic groups (NC64A, PBI, and SAG). As expected, the analysis of strict distance conservation between pairs of SCCGs within viral taxonomic groups (Figure S5: Analysis of pairwise distance conservation within phylogenetic groups—Figure_S5.pdf) indicates higher degree of strict pairwise distance conservation at greater distances than the “all-genome” comparison. However, even in this case, evidence of strict distance conservation is completely lost in bins larger than the strict 14–15 kbp bins. The analysis between taxonomic groups shown in Figure S6: Analysis of pairwise distance conservation across phylogenetic groups—Figure_S6.pdf, mirrors the results presented in Figure 2.

#### 3.2.2. Results of Growing Bin Analysis or Pairwise Distances

**Figure 3:**
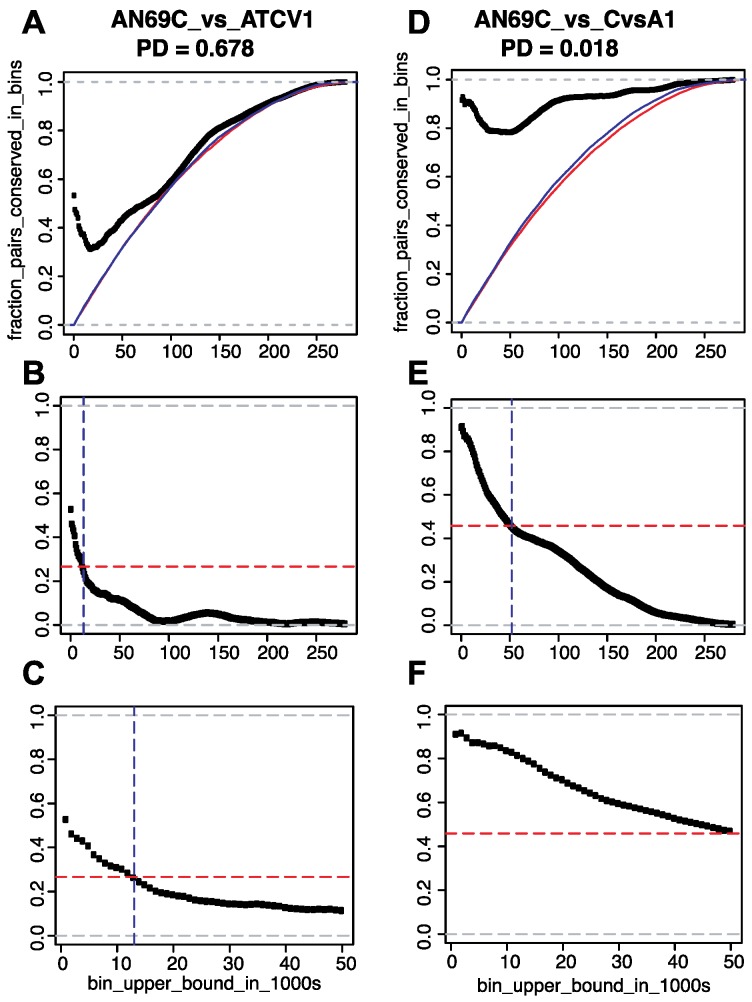
Analysis of pairwise conservation of SCCG distances in continuously increasing bin sizes. Representative analyses of two-genome comparisons of SCCG distance conservation in bins of growing size are presented. The distances between SCCGs in the genome of virus AN69C are compared against the genome of virus ATCV1 (large phylogenetic distance = 0.678) and those present in the virus CvsA1 (small phylogentic distance = 0.018). In all plots, the *x*-axes report the size of the bins considered in thousands of base pairs (e.g., point 1 represents a distance bin ranging from 0 to 1000 bp, point 5 represents a distance bin from 0 to 5000 bp, etc.). The *y*-axes in all plots represent the fraction of SCCG pairs whose distance is conserved within the same bin size in both genomes. Grey dashed lines represent upper and lower bounds of fractional conservation, 1 and 0, respectively. In (**A**,**D**) the *y*-axis reflects the calculated value of conservation arrived by direct comparison of the two genomes while in (**B**,**C**,**E**,**F**) the *y*-axis reports the degree of conservation minus the expected conservation based on a random gene shuffling model. (**A**) A direct comparison of SCCG distance conservation between viruses AN69C and ATCV1 (phylogenetic distance = 0.678) for bins of increasing size is depicted by the dotted black line. Models for expected conservation, based on a model of random shuffling derived from the respective cumulative distributions functions of pairwise gene distances in each genome, are drawn as red and blue lines for the first (AN69C) and second (ATCV1) genomes, respectively. Non-random conservation of pairwise distances appear as points above the random-shuffle models and approach the random model as bin sizes grow. (**B**) Plot of the data shown in (**A**) with the random model from the second genome subtracted. A horizontal dashed red line indicates the threshold of fractional conservation at which no “signal” of conservation can be reliably claimed if the random-shuffle model is applied. The vertical dashed blue line represents the intersection between black and red curves and represents the maximum bin size for which conservation of pairwise distances can be reliably reported. (**C**) A magnified portion of panel B. (**D**–**F**) The same analyses in (**A**–**C**) respectively, for the comparison of viruses AN69C and CvsA1 (phylogenetic distance = 0.018).

A second way of evaluating the conservation of pairwise distances between SCCGs is to ask: how often do pairs of SCCGs appear to stay within a maximum distance from one another? In this analysis, homologous pairs of genes are allowed to shuffle genomic positions with respect to one another in different genomes, but the homologs of the seed pair are always found within a size bin that does not exceed a threshold value. This question was addressed using the growing bin analysis and was conducted on all possible pairs of genomes. Illustrative examples are shown in Figure 3 and all comparisons are provided in File S7: All pairwise distance plots—File_S7.zip.

As shown in Figure 3A,D, this analysis also includes a comparison of SCCG distance conservation to that of a background model of gene shuffling in which all genes are allowed to shuffle independently and randomly in a way that preserves the distribution of naturally observed internal distances. This background model is represented by the cumulative distribution function of all pairwise distances between genes in a genome (Figure 3A,D). Any non-random conservation of distances is revealed by comparing the “random shuffle” model to the fraction of pairwise conserved distances observed in two genomes under comparison.

Genomic pairwise comparisons between virus AN69C and two other viruses (ATCV1 and CvsA1) illustrate the differences between closely and more distantly related genomes (Figure 3). In the comparison between two relatively distantly related genomes, we expect to see some conservation of pairwise distances at small bin sizes and relatively rapid convergence to the random model at larger bin sizes. Meanwhile, we expect to see conservation at small and large bin sizes in the comparison between two relatively closely related genomes. Both of these phenomena are observed and hold true across all comparisons for the evaluated chloroviruses (see: File S7). 

Subtracting the “random shuffle” background model from the computed conservation of distances between SCCGs (Figure 3B,C,E,F) helps one to visualize the non-random conservation of pairwise distances. Analyses of relatively distantly related genomes (Figure 3B,C) reveals a trend of relatively high non-random conservation of pairwise distance at small bin size, followed by a pattern of decreasing conservation as bin size increases. Ultimately, the point at which the degree of conservation approaches the background model can also be seen. By contrast, the subtraction of the “random shuffle” background from comparisons of relatively closely related genomes (Figure 3E,F) shows high conservation across nearly all bin sizes.

By applying the “random shuffle” model, we can estimate the bin size at which we can no longer reliably expect to see signals of non-random distance conservation. This point can be found by examining the half-maximal value of initial conservation and determining the associated bin size. These values are plotted in Figure 3C,E,F as dashed horizontal (half-maximal value) and dashed vertical (max bin size with non-random distance conservation) lines. As expected, relatively distantly related genomes show bin size thresholds of detectable distance conservation that are smaller than those derived from comparisons of closely related genomes. An analysis of all such computed thresholds suggests that, despite significant genomic shuffling in these genomes, selection may nevertheless constrain pairs of genes to distances between 0 and 15 kbp.

### 3.3. Identification of Gene Gangs

From the analyses discussed above, chloroviruses appear to have a significant preference for intergenic (center-to-center) distance conservation of SCCG pairs at intergenic (center-to-center) distances less than 15 kbp. Thus, we developed a technique (Figure S4) to detect “gene gangs,” which we define here as a collection of three or more genes that reside in close proximity to each other in all, or nearly all, of the 41 chloroviruses evaluated. The analyses expanded the set of genes searched from the SCCGs to include all annotated genes, with the exception of genes having 80 or more paralogs in the genomic dataset (see Materials and Methods). These seed clusters were then systematically analyzed using custom scripts and visualized in the context comparison tool JContextExplorer [5] (for more information, see Materials and Methods).

Gangs that have every member gene present in all 41 chloroviruses were referred to as “ruly gangs”. Gangs whose gene members were not perfectly conserved in all 41 *Chlorovirus* genomes were referred to as “unruly gangs.” Approximately a quarter (24.8%) of all genes with predicted CDSs are contained within ruly gene gangs, whereas about half (53.1%) of all core genes belonged to a ruly gene gang (Table 1). Five gene gangs were identified as ruly (complete cross-species conservation) (Table S9: Origin of unruly gang members—Table_S9.docx). By allowing for an unruliness index of approximately 0.9, 25 gene gangs were identified. Chlorovirus gene gangs varied in consensus number from 3 to 10 members (a few viruses have paralogous members within a gang, thus increasing the total count). Unruliness in one or more gangs was observed in all virus types.

We have considered that gene gang discovery could be influenced by the fact that the viral genomes were assembled from a varying number of contigs based on the assumption of collinearity to known reference genomes [4]. In many cases the assembly was disrupted due to repetitive sequences resulting in multiple contigs: 1–39 depending on the virus. It may be that the number of gene gangs discovered in the chloroviruses is underrepresented by this analysis, considering the varying number of unruly gangs per virus (1 to 11). However, this concern may not be warranted. There was no positive or negative correlation between the number of contigs to assemble a viral genome and the number of unruly gangs (as an indication of genome disruption) for any given virus (linear regression coefficient R^2^ = 0.005.) Thus, it appears that our method is efficient for gene gang discovery in the chloroviruses.

### 3.4. Topological Features of Selected Gene Gangs

**Figure 4:**
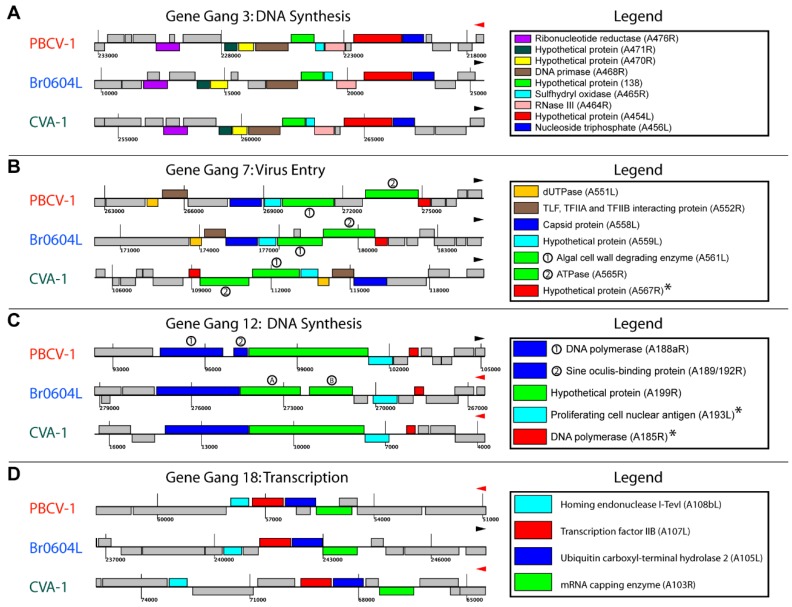
Topological features of four selected gene gangs (**A–D**). Genomic groupings are shown for a sample virus species associated with each host type (PBCV-1 from NC64A, Br0604L from SAG, and CVA-1 from Pbi). Gang members are indicated as colored genes, while surrounding non-gang member genes are shown in gray. Coordinates for each genome are given, as well as a black or red triangle to indicate increasing coordinate direction. In each genomic segment, genes above the center line are transcribed in the direction the triangle is pointing, while genes below the center line are transcribed in the direction opposite of the facing triangle. Each color refers to a different cross-species protein homology group. Annotations for each homology group, as well as the PBCV-1 gene ID, are provided in the legend to the right of each gene gang. If multiple paralogs were identified for a given homology group within a genomic grouping, paralogs are distinguished with circled numbers on the genomic groupings and legend (**B** and **C**). If a gene has been segmented into two parts by a probable insertion event or intron, the segmented parts of the gene are distinguished with a circled A and B (**C**). Annotations marked with no asterisk were associated in that genome for all 41 chloroviruses (ruly), while annotations marked with an asterisk were identified in 37 or more of the 41 chloroviruses, but not all 41 chloroviruses (unruly).

Gene gangs were observed dispersed across the viral genomes independent of virus type (e.g., Figure 1C). Visualization of individual gene gangs as they occur in specific viruses revealed diverse patterns of gene insertion, deletion, and rearrangement within gene gangs (Figure 4 and File S8: Genomic contexts for unruly gene gangs—File_S8.zip).

Members of certain gene gangs were separated by insertions (e.g., Figure 4b,d), and members within a gene gang may be inverted, re-located, and/or switch strands (Figure 4b). Alternatively, we suggest that a limited number of gangs may be under such stringent evolutionary pressure to avoid nearly all such modifications across species (e.g., Figure 4d).

The majority of gangs maintained perfect synteny (17 of 25) and those departing from this pattern were primarily virus type-specific (Table 2). However, strandedness was somewhat less conserved (13 of 25); again, deviations tended to be type specific. Interestingly, in two cases a subclade-specific deletion is observed, in NC64A-I (gene gangs 8 and 11). Otherwise, unruliness was not associated with specific subclades. All gangs were infiltrated by alien (non-gang) genes (3–41 viruses of any given gang were infiltrated). Remarkably, two chlorovirus gene gangs (19, 21) were perfectly conserved with respect to ruliness, synteny, and strandedness; and gene gang 2 had only a single deviation with the loss of one gene by the Pbi virus NE-JV-1 (Table 2). Given this one exception, chlorovirus gene gang 2, with eight consensus members, had the same gene orientation with respect to order and strand in all 41 viruses. However, all 41 viruses had alien genes in this gang (see File S8). Thus, it appears there is a strict requirement for topological conservation for this gang, but not for gene content.

Unruliness was not evenly distributed. Relatively few viruses had unruly gangs and these viruses tended to cluster in subclades of the virus types. *Chlorovirus* NE-JV-1 (subclade Pbi-I) was the most unruly virus of the 41 viruses analyzed, as inferred by dot-plot analyses [4] and most all other analyses of the genome structure, showing some lack of gene conservation in six gangs (Table S9). The Pbi-IIA subclade was the least disturbed with respect to ruliness, with only two gangs representing two viruses. Subclade SAG-II had the greatest number of unruly gang members (7) with 11 virus isolates.

### 3.5. Functional Evaluation of Gene Gangs

Of the 41 chloroviruses evaluated, only *Chlorovirus* PBCV-1 has been studied extensively [2]. Approximately 40% of the genes in gene gangs have no known or predicted function (i.e., hypothetical protein) for PBCV-1. However, 60% of the genes can be assigned with a functional annotation, with varying degrees of confidence (see Figure S10 and Table S1). There was a significant preference for genes functionally annotated in the categories associated with DNA replication, recombination and repair, and transcription (Figure S10). Approximately half (25 of 49) of the genes with these annotations in PBCV-1 were featured in gene gangs, suggesting that at least among the 41 chloroviruses evaluated, many genes associated with these functions tend to be selected as gene gangs. 

Additionally, the virion proteome [11] and transcriptome data [13] were compared with the annotations for the PBCV-1 genes in gene gangs (Table S1). The PBCV-1 gene gang members were mixed with respect to virion association, as well as gene expression patterns. So-called “late genes” (expressed after DNA synthesis begins) tend to code for proteins associated with the virus particle. Most of the late genes (75% of 81 genes) code for virion-associated proteins, and approximately half (48%) are gene gang members (Table S1).

A general problem plaguing chlorovirus genomics data is the lack of functional gene annotations: about 55% of the proteins are annotated as “hypothetical proteins” (however, this is not a problem unique to chloroviruses). In several chlorovirus gene gangs, the hypothetical protein genes co-occur with genes encoding proteins of known or probable functions. This suggests a path to better understanding the functions for unannotated genes through the concept of “guilt-by-association” [17]. This principle has been applied to genes in operons [18] with mixed results [19], however when the operon remains conserved across a set of organisms, annotation by context association tends to be more accurate [20,21]. We applied this technique to four selected gangs (Figure 4, from which general functional annotations for 10 protein families of unannotated function were predicted.) Thus, general functions for the gangs were inferred: gene gang 3, DNA synthesis (Figure 4A); gene gang 7, virus entry (Figure 4B); gene gang 12, DNA synthesis (Figure 4C); and gene gang 18, transcription (Figure 4D).

**Figure 5:**
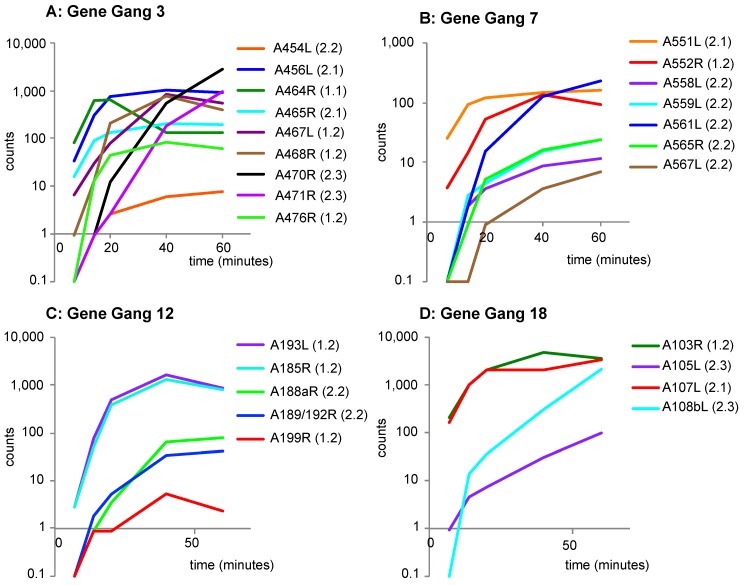
Transcriptional activation patterns of PBCV-1 genes from selected gangs. Each panel (**A–D**) reports the expression of genes in a single gene gang. Normalized sequence reads for multiple time points post infection (pi) were extracted from Blanc et al. (2014) [13] (see Table S1). The transcriptional activation patterns are noted next to the gene tag in parenthesis. The four selected gene gangs are the same as those used in Figure 4, and the color-coding of the annotated gene matches that in Figure 4. A value of 0.1 was given to normalize sequence read values of zero to allow for log_10_ plotting.

The transcriptional activation patterns for each of these four gene gangs from PBCV-1 is seen in Figure 5; the color codes of genes are matched between these data sets. All PBCV-1 genes are monocistronic with respect to gene expression [13]. When comparing expression patterns of gene gang members, it was apparent that the transcriptional activation of individual members had differential activation patterns when sampled at 7–60 min post infection. For example, gene gang 3 had approximately an 80-fold difference in transcript abundance at 7 min post infection when comparing CDSs A476R (ribonucleotide reductase) and A464R (RNase III). Likewise, gene gang 18 had an approximately 200-fold difference in transcript abundance at 7 min post infection when comparing CDS A108bL (homing endonuclease) to A103R (mRNA capping enzyme) and A107L (transcription factor IIB). In general, PBCV-1 gene gangs were comprised of both early and late genes, characterized by the expression profile parameter RNA-seq K-mean cluster [13]. However, gene gangs 2, 5, 9, 10, and 16 were predominately dominated by late expressing genes; whereas, gene gangs 6 and 22 had early expressing genes (Table S1). It is worth noting that the large, highly ruly, and most syntenic gang (chlorovirus gene gang 2 with eight consensus members) showed the same transcriptional pattern globally (all late expression) within the gang (RNA-seq K-means square cluster was 2.2 with one exception). Although the transcriptional pattern is consistent within this gang, individual transcripts vary significantly in abundance and do not correlate to strandedness. Five of the eight encoded proteins are associated with the virion [11], including at least one associated with the major capsid protein [22]. Thus, gene gang 2 appears to be highly coordinated temporally, constrained structurally and functionally, and potentially contributing to the virion maturation and release (note, the PBCV-1 A215L protein is an alkaline alginate lyase capable of digesting the algal cell wall that is not virion-associated [23,24]).

We originally coined the term “gene gang” to imply that the gene set functions cooperatively, and that each member carries out some function that contributes to the overall function of the gang. The inferred function of the gang may then contribute to the further understanding of the gang members: knowing what biochemical function one gene carries out may (or may not) help with predicting the function of a second gene, but when there is a larger set of genes with similar known or probable functions then it is possible to postulate the biochemical function of an hypothetical protein. This is the “guilt-by-association” assignment from being a member of a gang [17]. However, to begin this process, we need a “snitch”.

Although it was not possible to infer functionality for all of the chlorovirus gene gangs, some gangs were more transparent as to the putative function because they contain snitches. For example, PBCV-1 gene gang 12 (genome map positions 94,548–102,968) was populated by two DNA polymerase proteins and a sliding clamp/proliferating cell nuclear antigen (PCNA), proteins known to be involved in DNA synthesis (see Table S1). Additionally, the sine oculis-binding protein contains a nuclear localization domain. Together, these four proteins place the function of this gang in the realm of DNA synthesis, presumably viral DNA synthesis (the host cellular DNA is rapidly degraded upon infection [25]). The fifth member of this gang, A199R, has no known or probable function (hypothetical protein), but we infer that this protein contributes to viral DNA synthesis, although its precise activity remains unknown. Likewise, gene gang 17 (PBCV-1 genome map position 215,534–218,380) is likely involved in the regulation of gene expression, because three of the four members have known or probable functions contributing to cell signaling and the fourth member is an mRNA triphosphatase involved in 5′-capping [26]. Information for all of the PBCV-1 gene gang members is compiled in Table S1.

A gene gang may be associated with direct activity in a biochemical pathway, but it is also possible that certain members of a gang contribute through regulatory functions. For example, four gangs have a member whose known or probable function is a transcriptional regulator (either a transcription initiation factor, trans-activator, or antagonist to the TATA-containing promoters). Other gene gang members appear to be contributing through posttranslational modifications, as in signal transduction factors including a tyrosine protein phosphatase, dual specificity phosphatase, and two genes code for Rio2 serine protein kinase C.

Thus, the PBCV-1 gene gangs (and by inference the chlorovirus gene gangs) represent conserved clusters of collinear monocistronic genes with complex expression patterns and a multitude of known and probable functions.

## 4. Discussion

Here we examined 41 viruses in the genus *Chlorovirus*, and quantitatively characterized gene order conservation, genomic mobility, and positional stability. At the genome level, gene order was highly conserved among viruses that infect the same host alga, with only a few readily identifiable localized rearrangements, including inversions and indels. In contrast, at the genome level the gene synteny among viruses that infect different hosts was scrambled [4]. However, it was surprising to discover that a significant number of chlorovirus genes maintained a local level of synteny when compared across the broad range of viruses. Further evaluation of these local syntenic units or local collinear gene sets revealed a novel genetic organization in the chloroviruses that, with the exception of bacterial operons [27], uber-operons, and regulons [28], has not been described for any other virus or organism (to the best of our knowledge). We call these genetic elements “gene gangs”. We define gene gangs as a collection of genes that are all monocistronic genes and reside in close proximity to each other in the genomes investigated with at least three different protein families, with varying degrees of ruliness. Thus, in contrast to operons, gene gangs are a group of genes whose expression appears not to be coordinated by an operator, yet are presumed to be co-dependent.

A similar genetic organization in bacteria are observed, referred to as “gene teams” [29] (a term originally coined by Luc et al. [30]). A gene team is a set of genes that remain spatially close in a given set of genomes regardless of gene order. Using the method of Ling et al. formation of a gene team depends on the parameters “minsize,” the number of genes in each team, and “maxgap,” the maximum distance in nucleotides between any pair of adjacent genes within a gene team. For cases where many genomes are considered, an additional parameter, “minsupp”, may be varied, so that a cluster does not need to be conserved in all input genomes. The authors demonstrate that through a series of “decompose” and “filter” steps, they are able to generate gene teams in a computationally efficient manner.

The key distinction between the “gene gang” approach and the “gene team” approach is the handling of genes that are not present in all genomes. In the “gene team” approach, all genes are subjected to the “minsupp” parameter in a binary pass/fail manner. In the “gene gang” approach, we offer a similar parameter we call “ruliness,” however this parameter is subtly different from “minsupp”: when forming gene gangs, “cluster gangs” are first formed from “seed clusters,” which must be present in all genomes examined. The “ruliness” parameter is applied only to non-seed clusters.

The choice to identify and treat “seed genes” differently from the set of all genes is intentional: this way, we are able to simultaneously require perfect conservation (in the seed genes) and allow for some genes to be absent in some genomes (represented as the ruliness parameter). Our perfect conservation requirement is consistent with our objective to understand the evolutionary history and functional behavior of genes in the chloroviruses as a single, cohesive set of genomes, which requires that we consider genes that are representative of all of the chloroviruses (and are therefore ubiquitous). However, if functions change for genes nearby to these representative genes, genomic deletion or relocation events may occur, which we would like to detect without compromising the integrity of our set of representatives. Our “gene gangs” approach allows for this type of analysis, while the “gene teams” approach does not. A minor difference between the “gene teams” and “gene gangs” approach is how adjacent genomic distance is measured: in the “gene teams” approach, the gap between adjacent genes is used. In the “gene gangs” approach, the distance between adjacent gene centers is used.

Importantly, the discovery of gene gangs in chloroviruses and gene teams in certain bacteria indicate the concept of gene gangs/gene teams is possibly wide-spread in biological systems.

Gene gangs occurred in chloroviruses significantly more often than would be expected randomly, suggesting the existence of a strong selective pressure that keeps these genes in close proximity to each other. The functional categories of gene members within gene gangs provide new insights as to each gang member’s individual function in the context of the overall function of the gang.

Genetic material is often organized into structures of a higher order than the gene: e.g., many organisms (primarily prokaryotes) are known to organize functionally related genes into structures called “operons,” which place the regulation of their expression under the control of a single promoter [27]. Genes may also be organized in structures larger than operons, such as regulons, modulons, and Uber-operons [28,31], where the genomic proximity of associated genes facilitates efficient coordinated expression in ways more complicated than simple co-expression from the same promoter. There are reports of genes coordinating across large genomic distances [32,33] or in the case of eukaryotes, across chromosomes [34]. Such higher-order structural organizations are often evolutionarily conserved; for example, a study examining analogous microbial operons across species found that genes existing in an operon in one species were more likely than random to also exist in an operon in a closely related species [35], and Uber-operons (a genomic structure reflecting groups of operons) has been found to have some conservation across microbial species [28].

Additionally, prokaryotes have been observed to conserve gene context across multiple genomes when evaluating for candidate orphan enzymes in specific metabolic pathways, referred to as genomic metabolons [36]. Genomic metabolons appear to be associated with known operons; e.g., RegulonDB is shared by at least two genes in 74% of the *E. coli* K-12 metabolons. However, viruses tend not to recapitulate complete biosynthetic pathways, but rather tap into existing cellular metabolism by augmenting pathways in such a way as to favor the outcome for virus replication and success. Thus, it appears that genomic metabolons and gene gangs are very similar with respect to being conserved collinear gene clusters, yet are targeted at varying levels of the cellular biosynthetic hierarchy, and this suggests that gene gangs as observed in the chloroviruses may be present in organisms, as well as viruses.

Why do some genes remain conserved in clusters between species? A number of explanations have been provided over the years: genes may cluster according to the “selfish operon model”, which posits that gene clusters persist because proximity of the genes facilitates their collective transfer between species [37]. Another model used to explain gene clusters is the persistence model [38], which hypothesizes that by occupying less space, clustered genes are less likely to be disrupted by DNA deletion, insertion, and rearrangement events. This hypothesis bears similarity to the hypothesis that genes may be preserved in an operon together because they respond to the same combination of regulatory signals [39,40]. A number of models have been proposed to explain gene clustering owing to a fitness advantage, either by increased gene amplification, decreased diffusion times, or other metabolic arguments [41]

We believe the significance of this study lies in the fact that, the organization of biological information into units of a scale larger than a gene has received considerable attention in archaeal and bacterial species, however less research has addressed this issue in the context of large DNA viruses. Our discovery of multiple-gene biological structures conserved across a large set of phylogenetically diverse viruses suggests that such structures may be present in other viral genomes, and, if so, they may contribute to our understanding of viral genome construction and evolution. The comparative genomic approaches we outline and apply here should have wide applicability to other phylogenetically diverse genome sets.

## Figures and Tables

**Table 1:** Percentage of chlorovirus genes in gene gangs. Percentage of chlorovirus genes contained in gene gangs as a function of ruliness. Core genes were much more likely to exist in gene gangs than genes at large, which may reflect that the same evolutionary forces working to conserve genes may also act to spatially constrain them.

Genes of Interest	Ruliness						
	0.826	0.854	0.902	0.927	0.951	0.976	1.00
Core Genes	66.9%	65.5%	63.5%	60.4%	60.4%	57.7%	53.1%
All Genes	35.2%	33.7%	31.5%	29.3%	29.0%	27.7%	24.8%

**Table 2:** Chlorovirus gene gangs—conservation of gene content, synteny, strandedness, and the frequency of alien gene infiltration.

Gang	Maximum Number of Gang Members	Consensus Number of Gene Members of the Gang in Block Diagram ^$^	Number of Viruses That Are Ruly (100% Conserved in Gene Content), Total = 41	Number of Viruses That Maintain Synteny, Total = 41	Number of Viruses with Strand Conservation, Total = 41	Number of Viruses with Infiltrated Non-Gang (Alien) Genes, Total = 41
1	11	10	38	41	39	41
2	9	8	40	40	40	41
3	9	9	41	41	27	38
4	8	8	38	27	27 **^&^**	17
5 *	7	6	37	Virus type-specific	Virus type-specific	23
6	6	6	34	40	40	41
7	7	7	40	28	28	21
8	6	6	35	27	27	25
9	6	5	39	39	28	37
10	5	5	33	33	33	3
11	5	5	34	34	34	41
12	5	4	40	40	40	35
13	4	4	40	40	27	13
14	4	4	35	35	35	38
15	4	4	34	27	27	30
16	4	4	35	35	35	26
17	4	4	41	27	27	35
18	4	4	41	41 **^#^**	28	28
19	3	3	41	41	41	24
20	3	3	39	39	39	9
21	3	3	41	41	41	16
22	3	3	39	39	39	11
23	3	3	29	29	29	37
24	3	3	34	34	34	37
25	3	3	36	36	36	26

^$^ Generally, gene duplication is seen at a low frequency in various gangs causing the number of members to be greater than the consensus number. ^&^ There are two types of strand conservation in gene gang 4. One type is specific to NC64A and Pbi viruses; the other type is a multi-gene inversion specific to SAG viruses. * In gene gang 5 the gene content, synteny, and strandedness are specific to the virus type, but are conserved within the virus type. ^#^ In gene gang 18 all viruses have identical synteny but the SAG viruses are inverted relative to the NC64A and Pbi viruses.

## Supplementary Materials

The following are available online at http://www.mdpi.com/1999-4915/10/10/576/s1, Table S1: Comprehensive table of gene annotations. File S2: Newick formatted phylogeny of chloroviruses. File S3: Scripts used to calculate gene gangs. Figure S4: An overview of the method used to determine gene gangs. Figure S5: Analysis of pairwise distance conservation within phylogenetic groups. Figure S6: Analysis of pairwise distance conservation across phylogenetic groups. File S7: All pairwise distance plots. File S8: Genomic contexts for unruly gene gangs. Table S9: Origin of unruly gang members. Figure S10: Probable functional group distribution of PBCV-1 gene gang members.
