# Supplementary material for: Gene Gangs of the Chloroviruses: Conserved Clusters of Collinear Monocistronic Genes
Source: Viruses. 2018 Oct 20;10(10):576. doi: 10.3390/v10100576 (PMC6213493; doi:10.3390/v10100576)
Supplement: Supplementary file 1 [file viruses-10-00576-s001.zip › viruses-363410-suppl_/supplementary/Details of supplementary materials.docx]

Table S1: Comprehensive table of gene annotations. This table contains a comprehensive collection of information regarding each gene identified as belonging to a gene gang. Provided data include, chlorovirus ClusterID, PBCV-1 gene tag, PBCV-1 genome coordinates, accession number, inferred function, information from RefSeq, conserved domain information, SCOP70 annotations, and transcriptional and protein abundance data from previously reported studies. File S2: Newick formatted phylogeny of chloroviruses. A Newick formatted file of the chlorovirus phylogeny presented in Jeanniard et al. (2013) [4] and used for phylogenetic distance calculations in this study. File S3: Scripts used to calculate gene gangs. Gene gangs were identified using a set of scripts written in Java. The scripts are included in this zip archive. Figure S4: An overview of the method used to determine gene gangs. (A) Seed clusters—clusters that were universally or near-universally conserved—were extracted from each of the 41 chloroviruses, and the gene content found within a small genomic window centered on each of these genes were examined for the frequent presence of additional core genes occurring in the collinear window. (B) These “cluster gangs”—gangs calculated from a specific seed cluster—were amalgamated into non-cluster-centric gangs, yielding gene gangs. (C) Gene gangs were evaluated for ruliness and unruliness based on the presence or absence of certain gene members from the amalgamated group. Ruliness was evaluated from a range of 0.829 (present in 82.9% or more of genomic instances of a particular gang) to 1.00 (present in 100% of genomic instances of a particular gang). In certain cases, the ruliness had a major effect on the size of the gang (example shown in (C)), in other cases, varying the ruliness did not alter the identity of the gene gang (example not shown). Figure S5: Analysis of pairwise distance conservation within phylogenetic groups. Plots of pairwise distance conservation, as calculated in Materials and Methods, between SCCGs in different subsets of the chlorovirus genomes. In Figure S5 we compare the pairwise distance conservation of genes within the more closely related viral clades—(B) PBI viruses; (C) NC64A viruses; and (D) SAG viruses—to that of the same analysis over (A) all available genomes (also Figure 2). The x-axis indicates the distance bin evaluated while the y-axis reports the percent conservation of pairwise distances within said bin in the other genomes of each respective comparison set. The calculations were repeated four times allowing for different allowable percentage of genomic conservation (i.e., whether the pair met the distance criteria in either 100% (solid blue), 90% (dashed red), 80% (dashed green), or 70% (dashed black) of the genomes in the set). This analysis shows the degree to which each phylogenetic group contributes to the overall pairwise distance conservation observed in Figure 2 and also provides insight into how conserved pairwise distances are within phylogenetic groups. Figure S6: Analysis of pairwise distance conservation across phylogenetic groups. Plots of pairwise distance conservation, as calculated in Materials and Methods, between SCCGs in different subsets of the chlorovirus genomes. In Figure S6 we compare the pairwise distance conservation of genes between viral clades. In effect, the analysis used to produce Figure 2 is repeated between genomes from just two of the viral clades. Panel legends indicated first the set of genomes used as seeds and the genomes that were searched (Seed_v_SearchSet). (A) Type NC64A_v_PBI viruses; (B) Type NC64A_v_SAG viruses; (C) Type PBI_v_NC64A viruses; (D) Type PBI_v_SAG viruses; (E) Type SAG_v_NC64A viruses; and (F) Type SAG_v_PBI viruses. The x-axis indicates the distance bin evaluated while the y-axis reports the percent conservation of pairwise distances within said bin in the other genomes of each respective comparison set. The calculations were repeated four times allowing for different allowable percentage of genomic conservation (i.e., whether the pair met the distance criteria in either 100% (solid blue), 90% (dashed red), 80% (dashed green) or 70% (dashed black) of the genomes in the set). This analysis shows the degree to which pairwise distance comparisons across phylogenetic group contribute to the overall pairwise distance conservation observed in Figure 2 and also provides insight into how conserved pairwise distances are between phylogenetic groups. File S7: All pairwise distance plots. The collection of all genomic pairwise analyses of conservation of pairwise distances between SCCGs in growing bins. Representative figures are reported in Figure 3. For each file, the two genomes being compared are listed in the figure title along with the calculated phylogenetic distance between the two genomes. In all plots, the x-axes report the size of the bins considered in 1000s of base pairs (e.g., point 1 represents a distance bin ranging from 0 to 1000 bp, point 5 represents a distance bin from 0 to 5000 bp, etc.). The y-axes in all plots represent the fraction of pairwise distance conservation minus the expected conservation based on a random gene shuffling model that is derived from the respective cumulative distribution functions of pairwise gene distances in each genome. A horizontal dashed red line indicates the threshold of fractional conservation at which no “signal” of conservation can be reliably claimed if the random-shuffle model is applied. The vertical dashed blue line represents the intersection between black and red curves and represents the maximum bin size for which conservation of pairwise distances can be reliably reported. File S8: Genomic contexts for unruly gene gangs. Graphical representations for each unruly gene gang in each genome for which it is found. Table S9: Origin of unruly gang members. This table reports the identity and phylogenetic grouping of where each gene gang is found to be unruly. Virus clades include NC64A-1, NC64A-II, Pbi-I, Pbi-IIA, Pbi-IIB, SAG-1, and SAG-II as derived in [4]. Values of ruliness are color coded with green indicating that a gene gang is completely ruly in a clade and yellow, light orange, dark orange, and red shading, indicating increasing amounts of unruliness, respectively. Figure S10: Probable functional group distribution of PBCV-1 gene gang members. The *Chlorovirus* PBCV-1 gene gang members were annotated for probable functions, as compiled in Table S1. Using both sequence-matching and structure-inferred databases, a probable function was derived. These probable functions were clustered. The percent of genes determined to be within a cluster was calculated, *n* = 129 genes total for PBCV-1 gene gangs.
