# Supplementary material for: Gene Gangs of the Chloroviruses: Conserved Clusters of Collinear Monocistronic Genes
Source: Viruses. 2018 Oct 20;10(10):576. doi: 10.3390/v10100576 (PMC6213493; doi:10.3390/v10100576)
Supplement: Supplementary file 1 [file viruses-10-00576-s001.zip › viruses-363410-suppl_/supplementary/Figure_S5.pdf]

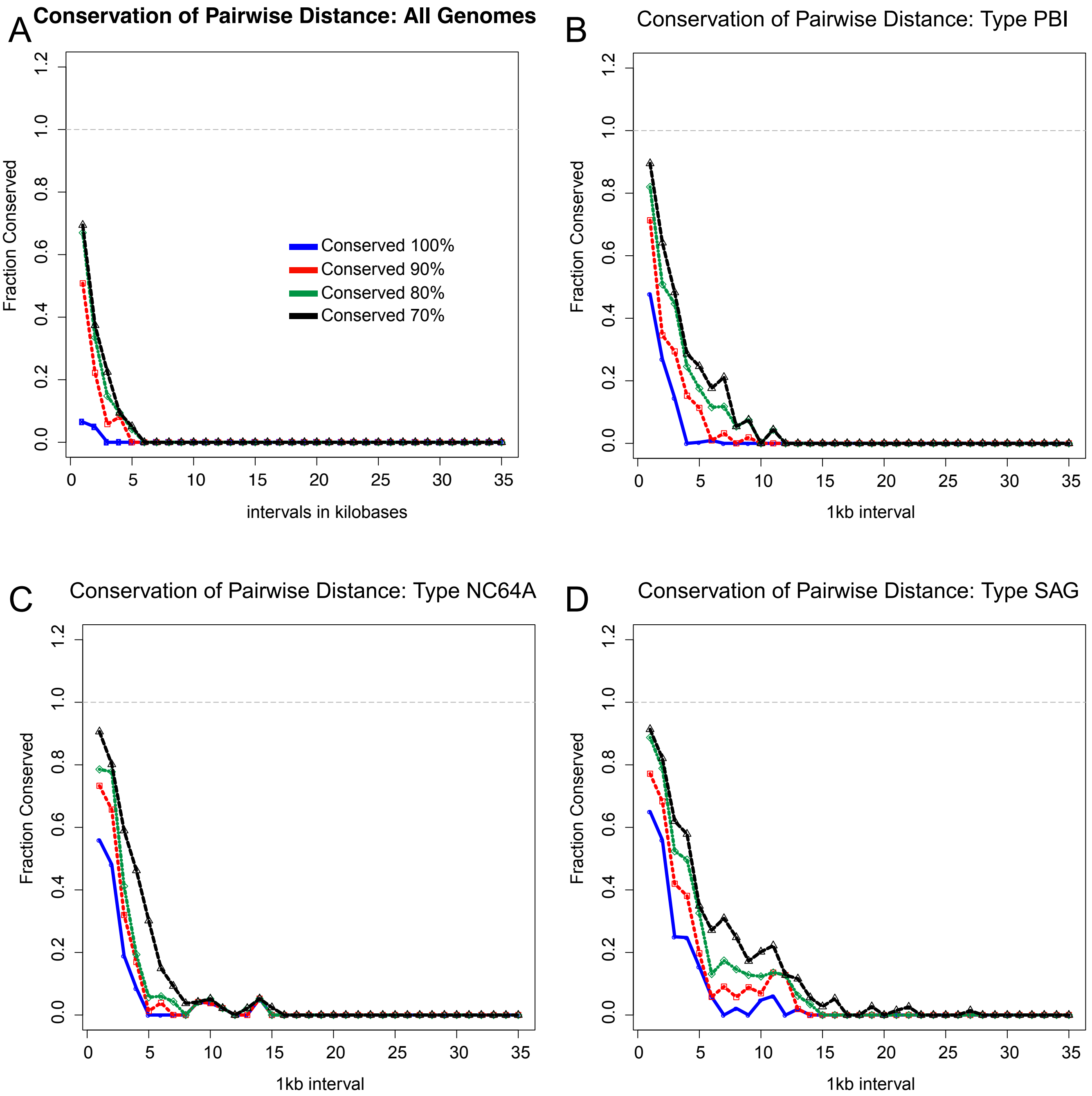

A
Conservation of Pairwise Distance: All Genomes
Fraction Conserved
intervals in kilobases
Conserved 100%
Conserved 90%
Conserved 80%
Conserved 70%
B
Conservation of Pairwise Distance: Type PBI
Fraction Conserved
1kb interval
C
Conservation of Pairwise Distance: Type NC64A
Fraction Conserved
1kb interval
D
Conservation of Pairwise Distance: Type SAG
Fraction Conserved
1kb interval
0.0
0.2
0.4
0.6
0.8
1.0
1.2
0
5
10
15
20
25
30
35
