# Supplementary material for: Gene Gangs of the Chloroviruses: Conserved Clusters of Collinear Monocistronic Genes
Source: Viruses. 2018 Oct 20;10(10):576. doi: 10.3390/v10100576 (PMC6213493; doi:10.3390/v10100576)
Supplement: Supplementary file 1 [file viruses-10-00576-s001.zip › viruses-363410-suppl_/supplementary/Table_S9.docx]

|  | |  |  | **Fraction of viruses in the *Chlorovirus* phylogenic clades that are unruly (0 = 100% ruliness)** | | | | | | | | | | | | | | | | | |  | | | | | |  |  |  |  |  |
| --- | --- | --- | --- | --- | --- | --- | --- | --- | --- | --- | --- | --- | --- | --- | --- | --- | --- | --- | --- | --- | --- | --- | --- | --- | --- | --- | --- | --- | --- | --- | --- | --- |
|  | |  |  |  |  |  |  |  |  |  |  |  |  |  |  |  |  |  |  |  |  |  | | | | | |  |  |  |  |  |
|  | |  |  | **Chlorovirus phylogenetic clades (derived from [4])** | | | | | | | | | | | | | | | | | |  | | | | | |  |  |  |  |  |
|  | |  | **Virus type-Clade** | **NC64A-I** | **NC64A-II** | | **Pbi-I** | | **Pbi-IIA** | | **Pbi-IIB** | | | **SAG-I** | | | **SAG-II** | | | | | | |  | | | | | |  |  |  |
| **Gang number** | **Consensus number of gene members of the gang in block diagram%** | **Number of viruses in the clade** | **6** | **8** | | **1** | | **3** | | **10** | | | **2** | | | **11** | | | | | | | **Total fraction of unruly gangs in 41 viruses** | | | | | |  |  |  |  |
| 1 | | 10 |  | 0.00 | 0.43 | | 0.00 | | 0.00 | | 0.00 | | | 0.00 | | | 0.00 | | | | | | | 0.07 | | | | | |  |  |  |
| 2 | | 8 |  | 0.00 | 0.00 | | 1.00 | | 0.00 | | 0.00 | | | 0.00 | | | 0.00 | | | | | | | 0.02 | | | | | |  |  |  |
| 3 | | 9 |  | 0.00 | 0.00 | | 0.00 | | 0.00 | | 0.00 | | | 0.00 | | | 0.00 | | | | | | | 0.00 | | | | | |  |  |  |
| 4& | | 8 |  | 0.29 | 0.29 | | 0.00 | | 0.00 | | 0.00 | | | 0.00 | | | 0.00 | | | | | | | 0.10 | | | | | |  |  |  |
| 5* | | 7 |  | 0.57 | 0.00 | | 0.00 | | 0.00 | | 0.00 | | | 0.00 | | | 0.00 | | | | | | | 0.10 | | | | | |  |  |  |
| 6 | | 6 |  | 0.00 | 0.00 | | 1.00 | | 0.00 | | 0.00 | | | 1.00 | | | 0.36 | | | | | | | 0.17 | | | | | |  |  |  |
| 7 | | 7 |  | 0.00 | 0.00 | | 1.00 | | 0.00 | | 0.00 | | | 0.00 | | | 0.00 | | | | | | | 0.02 | | | | | |  |  |  |
| 8 | | 6 |  | 0.86 | 0.00 | | 0.00 | | 0.00 | | 0.00 | | | 0.00 | | | 0.00 | | | | | | | 0.15 | | | | | |  |  |  |
| 9 | | 5 |  | 0.29 | 0.00 | | 1.00 | | 0.00 | | 0.11 | | | 0.00 | | | 0.00 | | | | | | | 0.10 | | | | | |  |  |  |
| 10 | | 5 |  | 0.00 | 0.14 | | 1.00 | | 0.00 | | 0.00 | | | 0.50 | | | 0.45 | | | | | | | 0.20 | | | | | |  |  |  |
| 11 | | 5 |  | 0.86 | 0.00 | | 0.00 | | 0.00 | | 0.11 | | | 0.00 | | | 0.00 | | | | | | | 0.17 | | | | | |  |  |  |
| 12 | | 4 |  | 0.00 | 0.00 | | 0.00 | | 0.00 | | 0.11 | | | 0.00 | | | 0.00 | | | | | | | 0.02 | | | | | |  |  |  |
| 13 | | 4 |  | 0.00 | 0.00 | | 1.00 | | 0.00 | | 0.00 | | | 0.00 | | | 0.00 | | | | | | | 0.02 | | | | | |  |  |  |
| 14 | | 4 |  | 0.00 | 0.00 | | 0.00 | | 0.50 | | 0.33 | | | 0.00 | | | 0.09 | | | | | | | 0.15 | | | | | |  |  |  |
| 15 | | 4 |  | 0.00 | 0.00 | | 0.00 | | 0.00 | | 0.00 | | | 0.00 | | | 0.64 | | | | | | | 0.17 | | | | | |  |  |  |
| 16 | | 4 |  | 0.00 | 0.86 | | 0.00 | | 0.00 | | 0.00 | | | 0.00 | | | 0.00 | | | | | | | 0.15 | | | | | |  |  |  |
| 17 | | 4 |  | 0.00 | 0.00 | | 0.00 | | 0.00 | | 0.00 | | | 0.00 | | | 0.00 | | | | | | | 0.00 | | | | | |  |  |  |
| 18 | | 4 |  | 0.00 | 0.00 | | 0.00 | | 0.00 | | 0.00 | | | 0.00 | | | 0.00 | | | | | | | 0.00 | | | | | |  |  |  |
| 19 | | 3 |  | 0.00 | 0.00 | | 0.00 | | 0.00 | | 0.00 | | | 0.00 | | | 0.00 | | | | | | | 0.00 | | | | | |  |  |  |
| 20 | | 3 |  | 0.00 | 0.29 | | 0.00 | | 0.00 | | 0.00 | | | 0.00 | | | 0.00 | | | | | | | 0.05 | | | | | |  |  |  |
| 21 | | 3 |  | 0.00 | 0.00 | | 0.00 | | 0.00 | | 0.00 | | | 0.00 | | | 0.00 | | | | | | | 0.00 | | | | | |  |  |  |
| 22 | | 3 |  | 0.00 | 0.00 | | 0.00 | | 0.00 | | 0.00 | | | 0.00 | | | 0.18 | | | | | | | 0.05 | | | | | |  |  |  |
| 23 | | 3 |  | 0.00 | 0.00 | | 0.00 | | 0.75 | | 1.00 | | | 0.00 | | | 0.00 | | | | | | | 0.29 | | | | | |  |  |  |
| 24 | | 3 |  | 0.00 | 0.00 | | 0.00 | | 0.00 | | 0.00 | | | 0.00 | | | 0.64 | | | | | | | 0.17 | | | | | |  |  |  |
| 25 | | 3 |  | 0.00 | 0.00 | | 1.00 | | 0.25 | | 0.22 | | | 0.00 | | | 0.18 | | | | | | | 0.15 | | | | | |  |  |  |
|  | |  | **Number of unruly gangs in clade** | 5 | 5 | | 7 | | 3 | | 6 | | | 2 | | | 7 | | | | | | |  | | | | | |  |  |  |
|  | |  | **Fraction of gangs that are unruly** | 0.20 | 0.20 | | 0.28 | | 0.12 | | 0.24 | | | 0.08 | | | 0.28 | | | | | | |  | | | | | |  |  |  |
|  | |  |  |  |  | |  | |  | |  | | |  | | |  | | | | | | |  | | | | | |  |  |  |
|  | | **Host type** | **Chlorovirus phylogenetic clade** | **Chlorovirus** |  | |  | |  | |  | | |  | | |  | | | | | | |  | | | | | |  |  |  |
|  | | Chlorella variabilis NC64A | NC64A-I | NY2A, Nys-1, NY-2B, AR158, MA-1D, IL-5-2s1 | | | | | |  | |  | | |  | | | | | | |  | | | | | |  |  |  |  |  |
|  | |  | NC64A-II | PBCV1, AN69C, IL-3A, NE-JV-4, KS1B, CviKI, CvsA1, MA-1E | | | | | | | |  | | |  | | | | | | |  | | | | | |  |  |  |  |  |
|  | | Micratinium conductrix Pbi | Pbi-I | NE-JV-1 | | | |  | | | | |  | | |  | | |  | |  | |  | | |  | | | | | |  |
|  | |  | Pbi-IIA | Fr5L, CZ-2, OR0704.2.2 | |  | | | | | | | | |  | | |  | |  | |  | | |  | | | | | |  |  |
|  | |  | Pbi-IIB | MT325, Can18-4, CVM-1, AP110A, CVR-1, CVA-1, CVG-1, NW665.2, CVB-1, FR483 | | | | | | | | | | | | | | | | | |  | | | | | |  |  |  |  |  |
|  | | Chlorella heliozoeae SAG 3.83 | SAG-I | MN0810.1, Canal-1 | |  | | | | | | | | |  | | |  | | | |  | | |  | |  | | | | | |
|  | |  | SAG-II | GM0701.1, Br0604L, TN603.4.2, NTS-1, OR0704.3, Can0610SP, NE-JV-2, ATCV-1, NE-JV-3, WI0606, MO0605SPH | | | | | | | | | | | | | | | | | | | | | | | | |  |  |  |  |
