# Supplementary figures and images for: Gene Gangs of the Chloroviruses: Conserved Clusters of Collinear Monocistronic Genes
Source: Viruses. 2018 Oct 20;10(10):576. doi: 10.3390/v10100576 (PMC6213493; doi:10.3390/v10100576)

# Probable functional group distribution of PBCV-1 Gene Gang member, n=129

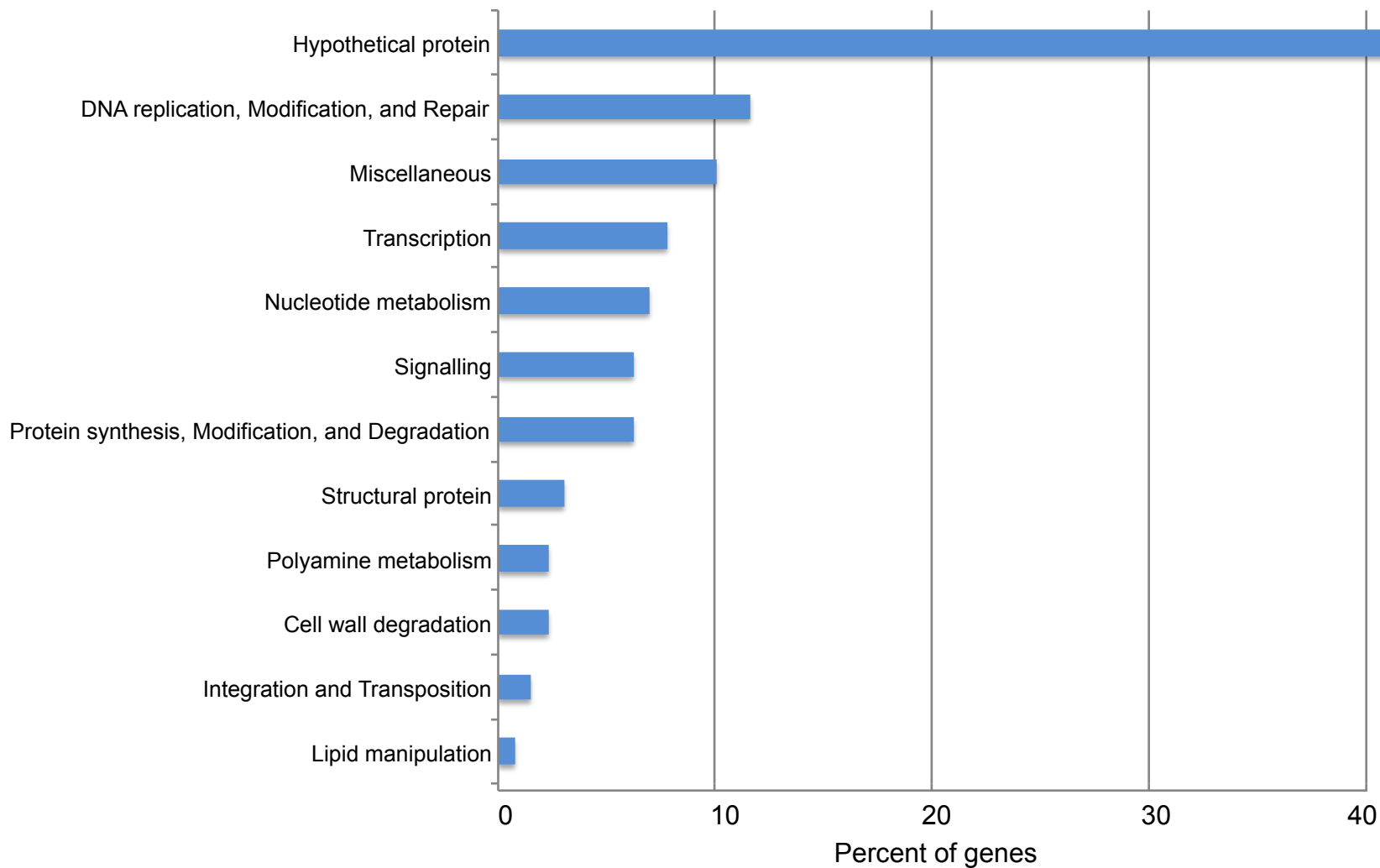

Supplement: Supplementary file 1 [file viruses-10-00576-s001.zip › viruses-363410-suppl_/supplementary/Figure S10.pdf]

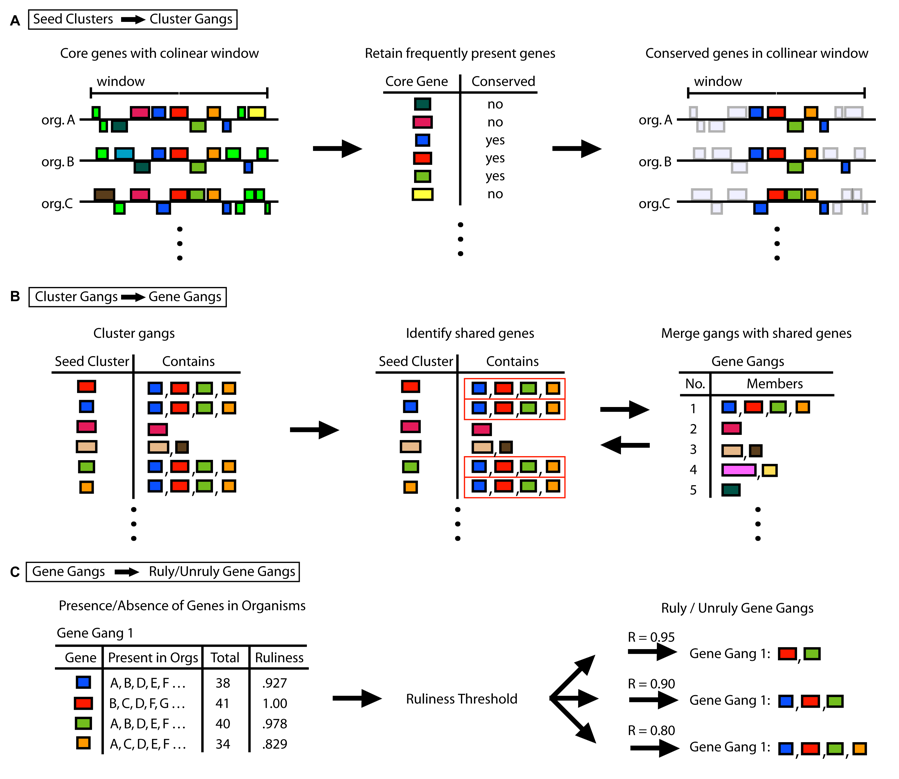

Supplement: Supplementary file 1 [file viruses-10-00576-s001.zip › viruses-363410-suppl_/supplementary/Figure_S4.png]
